# Supplementary material for: Investigation of the Seroprevalence of Brucella Antibodies and Characterization of Field Strains in Immunized Dairy Cows by B. abortus A19
Source: Vet Sci. 2024 Jun 28;11(7):288. doi: 10.3390/vetsci11070288 (PMC11281660; doi:10.3390/vetsci11070288)

The figures in the manuscript and full-length gels

（1）Figure S1. Development of a PCR method to identify Brucella A19 and non - A19 strains of the original Full-length gel. Original picture starting from left 3-8 lane corresponds to M and 1-5 in Figure 2.

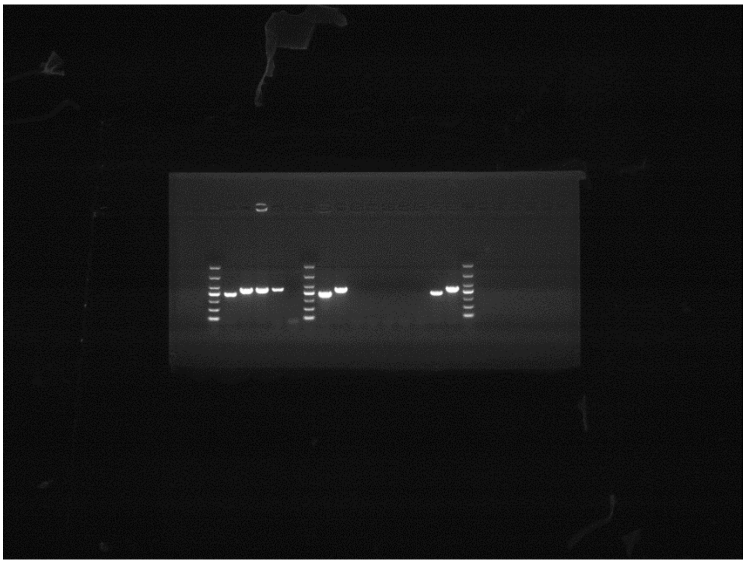

（2）Figure S2. Specificity of the PCR method for identification of Brucella A19 and non-A19 strains of the original Full-length gel. Original picture starting from left 9-16 lane corresponds to M and 1-7 in Figure 3.

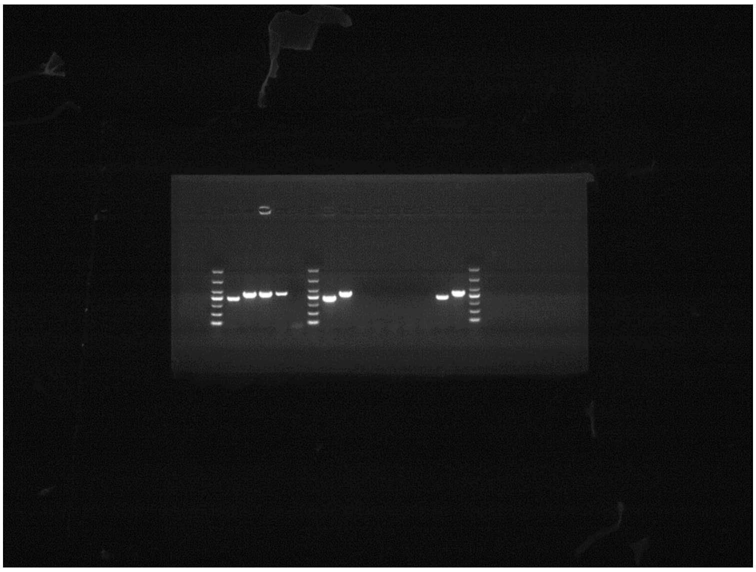

（3）Figure S3. Sensitivity of the PCR method for identification of Brucella A19 and non-A19 strains of the original Full-length gel. Up part of the gel in the original picture starts from the left numbers 2-8 and 15-22 lane correspond to 1-7 and M-14 in Figure 4.

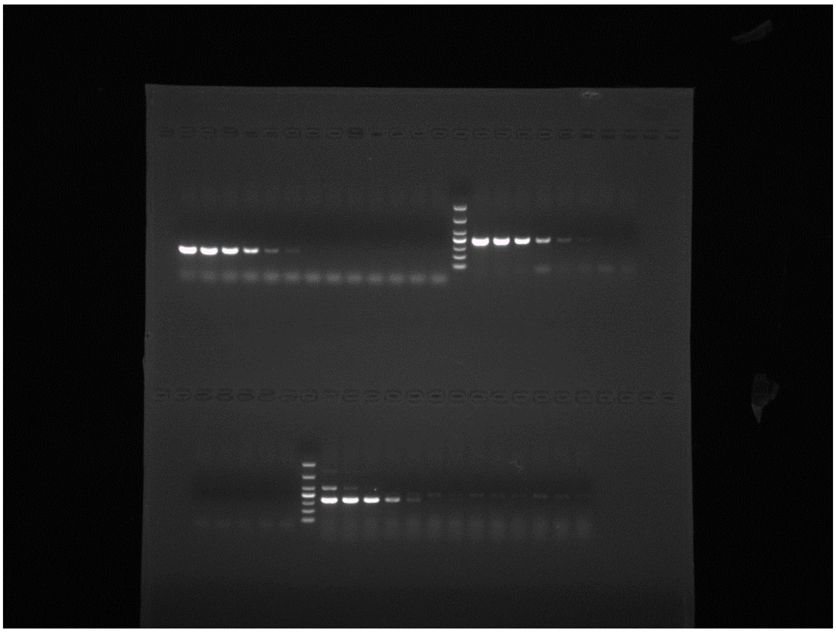

（4）Figure S4. Investigation of Brucella field strains in 10 large-scale dairy farms using the established PCR method of the original Full-length gel. Up part of the gel in the original picture starts from the left and numbers 4-17 lane correspond to M and 1-13 in Figure 5.

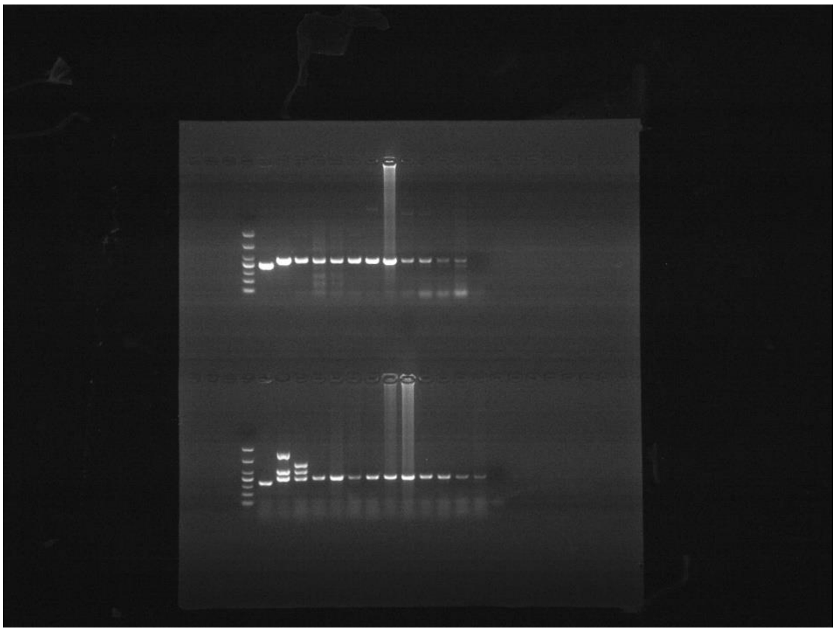

（5）Figure S5. The identification of Brucella field strain species of the original Full-length gel. Down part of the gel in the original picture starts from the left and numbers 6-22 lane correspond to M -15 in Figure 6.

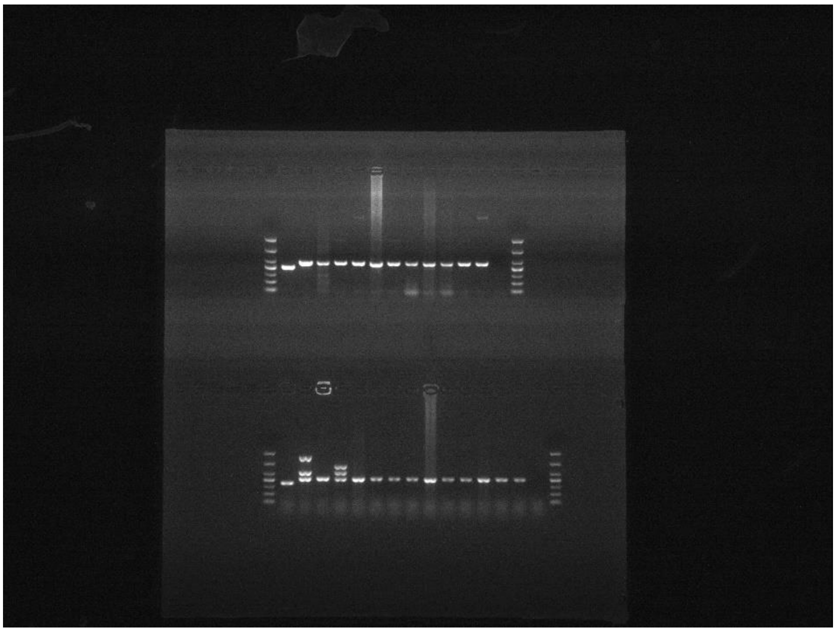

Supplement: Supplementary file 1 [file vetsci-11-00288-s001.zip › vetsci-2971106-supplementary.pdf]
